# Stated Preferences for Dog Characteristics and Sources of Acquisition

**DOI:** 10.3390/ani7080059

**Published:** 2017-08-05

**Authors:** Courtney Bir, Nicole J. Olynk Widmar, Candace C. Croney

**Affiliations:** 1 Department of Agricultural Economics, Purdue University, West Lafayette, IN 47907, USA; nwidmar@purdue.edu; 2 Department of Comparative Pathobiology, Purdue University, West Lafayette, IN 47907, USA; ccroney@purdue.edu

**Keywords:** dog acquisition, public perception, best-worst, preferences

## Abstract

**Simple Summary:**

How people choose to acquire dogs and the characteristics they seek may provide insight into perceptions of ethical and socially responsible dog ownership, as acquiring a dog is the first step in dog ownership. Even if one does not intend to acquire a dog, perceptions of acquisition methods may impact views of pet industries and/or voting behaviors related to animal welfare regulation. This analysis (1) investigates the relationships between U.S. resident demographics and their level of agreement regarding statements related to dog acquisition and welfare considerations, (2) analyzes the relationships between U.S. resident demographics and their views on dog characteristics, and (3) analyzes U.S. residents’ relative ranking (in terms of most ethical) ways to acquire a dog. The findings of the current study affirm that appearance, compatibility with owner lifestyles, previous experience, and cost inform people’s decisions about dog acquisition. The relative importance of these criteria varied across respondents; as has been previously observed, women favored rescue/adoption more than men. Our results collectively indicate the growing appeal of adopting dogs from shelters and rescue organizations for many respondent segments. Different segments of the companion animal industry and those with different dog interests may want to consider tailoring their education and/or marketing communications to these groups accordingly.

**Abstract:**

People’s preferences for where they acquire dogs and the characteristics they focus on may provide insight into their perceptions of socially responsible pet ownership, as acquiring a dog is the first step in dog ownership. An online survey of 1523 U.S. residents was used to aid understanding of public perceptions of dog acquisition. Likert-scale questions allowed respondents to assign a level of agreement, within the given scale, to ten statements related to dog acquisition. A significantly higher percentage of women (39.6%) than men (31.7%) agreed that the only responsible way to acquire a dog is through a shelter/rescue. More women (71.3%) than men (66.4%), as well as those with a higher household income (71%), identified source as important. Best-worst methodology was used to elicit perceptions regarding the most/least ethical ways to acquire a dog. Three subgroups were identified, one of which had an overwhelmingly large preference share (96%) for adoption. The second group had more evenly distributed preference shares amongst the various dog acquisition methods, while the third indicated a preference for “homeless” pets. Additional investigation of the values/beliefs underlying the preferences of these groups is necessary to design appropriately tailored companion animal-focused communication strategies for these different groups.

## 1. Introduction

Over 54 million U.S. households now include companion dogs [1]. Where or how people choose to acquire their dogs, and the characteristics they look for in the animals they select, may provide insight into perceptions about ethical and socially responsible pet ownership. Acquiring a dog is the first step in dog ownership; the acquisition method may be related to responsible pet ownership overall, but is only one of many aspects to be considered. Such perceptions, in turn, may have implications for dog breeders, various pet industry sectors, as well as animal protection and sheltering organizations. Dog acquisition is a complex subject which incorporates several aspects of public demand [2], ethics and a multitude of factors which govern and impact the relationships between human and non-human species. Beyond the impacts of dog acquisition on pet industries and animal-interested parties, dog ownership has become a topic of interest for health care professionals and health industries, as dogs have been found to enhance physical and mental health. For example, the physical and mental health of elderly people was improved by dog companionship, through a support network of dog owners, and decreased the need for health and social services [3].

When acquiring a dog, individuals are making complex decisions and are processing information from many, sometimes conflicting, sources. Although individuals do not always follow through (in action) with what they state as their preferences, it is nonetheless important to quantify these self-stated preferences, as they may reflect people’s actual or perceived values. Studies indicate high diversity in people’s preferred characteristics when selecting companion dogs [4] However, appearance is among the most consistently cited determinant of the decision to purchase or adopt a dog [2,5]. The dog’s behavior and temperament [2,6,7], size, breed, age, coat color, health and whether he or she is purebred, neutered or intact [6,8,9,10] also appear to be important to dog owners and potential adopters. 

In regard to rationales for people′s decision-making relative to obtaining dogs, Maddalena et al. [11] reported that for those who elected to obtain a dog from a shelter, desire to help dogs was a major determinant. For those who did not want a shelter dog, common reasons included wanting a purebred dog and being uncertain that a shelter could provide the type of dog they desired [11]. Concerns about the welfare of dogs originating from high-volume commercial breeding operations have led to several recent U.S. legislative proposals aimed at curtailing sales of dogs from pet stores which source dogs from such breeders [12,13]. Given the pressure for legislation to influence both dog breeding practices, and the sources from which U.S. residents can acquire dogs, quantifying public perceptions of those sources of acquisitions is necessary.

Although reasons for dog ownership are numerous and widely recognized, relatively few studies exist on the sources people use to obtain dogs, people’s stated or actual preferences, and the reasons underlying these choices. Such studies often only include dog owners. Even if one does not intend to acquire a dog, perceptions of various acquisition methods may impact views of pet industries and/or voting behaviors related to animal welfare regulation. This paper (1) analyzes the relationships between U.S. resident demographics and their level of agreement regarding statements related to dog acquisition, and resultant potential welfare considerations, (2) analyzes the relationships between U.S. resident demographics and their views on the most important dog characteristics, and (3) analyzes U.S. residents’ relative ranking (in terms of most ethical) ways to acquire a dog.

## 2. Materials and Methods

### 2.1. Survey Instrument and Data Collection

Qualtrics, an online survey tool, was used to gather information in October 2015, from 1523 U.S. residents. The survey was designed to collect basic demographic information as well as information regarding animal ownership, dog acquisition, dog welfare, and dog breeding. Additionally, questions specifically for dog-owning households, including ways that dogs were acquired and reasons dogs were acquired in that particular manner were included. University IRB (Internal Review Board) approval was obtained. Survey respondents were obtained through the use of a large opt-in panel database by Lightspeed GMI. The sample was targeted to be representative of the U.S. population. Proportions of respondents targeted in the sample with respect to gender, income, education, and geographical region of residence are reflective of the U.S. Census Bureau reported proportions for the U.S. population [14,15,16,17,18]. Regions of residence were used as defined as in the Census Bureau Regions and Divisions.(Regions were defined, according to the U.S. Census Bureau, as follows: Northeast includes Connecticut, Maine, Massachusetts, New Hampshire, Rhode Island, Vermont, New Jersey, New York, and Pennsylvania; Midwest includes Indiana, Illinois, Michigan, Ohio, Wisconsin, Iowa, Kansas, Minnesota, Missouri, Nebraska, North Dakota, and South Dakota; South includes Delaware, District of Columbia, Florida, Georgia, Maryland, North Carolina, South Carolina, Virginia, West Virginia, Alabama, Kentucky, Mississippi, Tennessee, Arkansas, Louisiana, Oklahoma, and Texas; and the West includes Arizona, Colorado, Idaho, New Mexico, Montana, Utah, Nevada, and Wyoming, Alaska, California, Hawaii, Oregon, and Washington.) Respondents were required to be 18 years of age or older to participate. Within the survey, a randomly selected subset of participants, *n* = 507, were selected to answer a best-worst choice experiment, focusing on respondent perception of the most and least ethical ways of acquiring a dog; That subsample of *n* = 507 is used exclusively in this analysis.

### 2.2. Summary Statistics and Cross-Tabulations

The subsample of responses, *n* = 507, was summarized by calculating the frequencies for categorical variables and means for the continuous variables. Analyses using cross-tabulations were completed to investigate the relationships between responses to questions pertaining to: dog welfare in relation to dog acquisition, pet ownership, important dog characteristics (in the opinion of the respondent), and demographics.

Likert-scale questions allowed respondents to assign a level of agreement, within the given scale of 1 (completely agree) to 7 (completely disagree), regarding ten statements related to dog acquisition. Likert-scale questions enable respondents to express their agreement in levels; for analysis purposes, selections of 1, 2, or 3 were condensed into “agree”, selections of 4 were labeled “neutral”, and selections of 5, 6, and 7 were condensed into “disagree”.

Within the responses to the demographic question “I am ____ years old” the age categories 18–24 and 25–34 were condensed into the age category 18–34, the age categories 35–44 and 45–54 were condensed into the age category 35–54, and the age categories 55–65 and 66–88 were condensed into the age category 55–88 to facilitate analysis and to more closely match analysis completed in previous literature which used broader categories such as young and old. The demographic question, “The best description of my educational background” was aggregated so that any respondents who did not indicate degree completion were in the category “Less than a college degree”, and anyone who completed at least a college degree were in the category “College degree or higher”. For the cross-tabulation analysis, note the percentages of each demographic add up to one hundred percent when summed across level of agreement for each statement, and the neutral category has been dropped from the presentation for brevity, but was incorporated in all statistical analysis for completeness.

Respondents were asked on a Likert scale from one to four, with one being very unimportant and four being very important to indicate, “What characteristics do you look for or believe are most important in acquiring a dog?” These included breed, appearance, compatibility with owner lifestyle, behavior, genetic health, physical health, cost, experience/reputation of source, and source of the dog. These factors were based on those reported as having varying levels of importance to current and prospective dog owners investigated in previous studies [2,6,8]. Responses of one and two were combined into unimportant and responses of three and four were combined into important. The responses were analyzed using cross-tabulations to determine if there was a relationship between the responses and demographics. 

### 2.3. Dog Acquisition Preferences via Best-Worst Scaling

Respondents were shown a series of best-worst scaling (BWS) choice scenarios and were asked to indicate the most and least ethical source to acquire a dog from a list of sources. Respondents were not given a definition of ethical in order to avoid biasing the respondent. The intention of this question is for respondents to use their personal definition and beliefs regarding ethical dog acquisition. Their personal beliefs are likely to impact how they might vote on dog acquisition legislature, and how they communicate with others regarding dog acquisition. Their responses were analyzed to elicit respondents′ preferences for the eight sources to acquire a dog included in this experiment. BWS forces the decision maker to make tradeoffs that more closely reflect actual choices [19,20]. To conduct the BWS experiment, respondents were asked to indicate, among eight different ways of acquiring a dog, which options they found the most and least ethical. The eight sources for acquiring dogs were chosen based on previous literature. Weiss et al. [21] included acquiring a dog from a friend, a shelter, and as a stray in their exploration of re-homing dogs and cats. Additionally, other common sources of dog acquisition, not well addressed in the literature were explored. In total, the eight dog acquisition paths studied in this analysis were: adoption, purchased directly from a breeder on site, online purchase directly from a breeder, purchased from an online retailer, purchased from a pet store, stray, gift from a friend or family member, and other. 

The BWS experiment was designed so that every participant was presented with eight blocks, each containing different combinations of seven of the eight ways to acquire a dog. The experimental design allowed each of the eight ways to acquire a dog to be selected between zero and seven times. An example best/worst block seen by respondents can be found in Table 1 The blocks presented included seven ways to acquire a dog (j). The respondents’ choices of the most ethical and least ethical ways to acquire a dog were used to determine the location along a continuum from most ethical to least ethical for each dog acquisition strategy. The location of the pathway to acquire a dog on the scale of most ethical way to acquire a dog is represented by $\lambda_{j}$. Thus, how ethical a respondent views a particular way to acquire a dog, which is unobservable to researchers, for respondent *i* is:

(1) $$I_{ij}=\lambda_{j}+\;{ℇ}_{ij}$$

where $\;{ℇ}_{ij}$ denotes a random error term. The probability that the respondent i chooses the way to acquire a dog j as the most ethical way to acquire a dog and the way to acquire a dog k as the least ethical way to acquire a dog is the probability that the difference between $I_{ij}$ and $I_{ik}$ is greater than all potential differences available from the choices presented to each survey respondent. Assuming the error term is independently and identically distributed type I extreme value, the probability of choosing a given most ethical-least ethical combination takes the multinomial logit form [19] represented by:

(2) $$Prob\left(j=best\;⋂\;k=worst\right)=\frac{e^{\lambda_{j}-\lambda_{k}}}{\sum_{l=1}^{J}\sum_{m=1}^{J}e^{\lambda_{l}-\lambda_{m}}-J}$$

Maximum likelihood estimation (MLE) is used to estimate the parameter $\lambda_{j}$ which represents how ethical attribute j is relative to the least ethical way to acquire a dog. The least ethical way to acquire a dog is determined through analysis of responses and its value must be normalized to zero to prevent multicollinearity [19].

A multinomial logit model (MNL), which allows for homogeneity among individuals, and a random parameters logit (RPL) model was specified to allow for continuous heterogeneity among individuals, following Lusk and Briggeman [19]. Using individual-specific parameter estimates from the RPL model, individual-specific preference shares were calculated.

The LCM (latent class model) allows respondents to be sorted into a specified number of classes with homogenous preferences within that class; however, across classes, preferences are heterogeneous [22]. Individual respondents are assigned to a latent class and parameters for each class are simultaneously estimated [23]. Given the respondent belongs to a specific latent class, denoted as *s*, the conditional probability of choices is represented as:

(3) $$\left(Prob\left(j=best\;⋂k=worst\right)|s\right)=\frac{e^{\lambda_{js}-\lambda_{ks}}}{\sum_{l=1}^{J}\sum_{m=1}^{J}e^{\lambda_{ls}-\lambda_{ms}}-J}$$

where the $\;\lambda_{js}$ and $\lambda_{ks}$ parameters are class specific [24]. These classes are unobservable and the probability of membership in a class takes the multinomial logit form:

(4) $$Prob\left(s\right)=\frac{e^{\left(\theta_{s}Zk\right)}}{\sum_{s=1}^{S}e^{\theta_{s}Zk}}$$

where Zk is a set of hypothesized drivers of class membership and $\theta_{s}$ is a parameter vector that is normalized to zero that characterizes the impact the drivers have on class membership [24]. Parameter estimates are not plainly interpretable; preference shares provide a more intuitive means of analyzing relationships between the attributes than the coefficient estimates [25]. The shares of preferences are calculated as:

(5) $$share_{j}=\frac{e^{\lambda_{j}}}{\sum_{k=1}^{J}e^{\lambda_{k}}}$$

The shares must sum to one across the eight attributes studied within each class. The calculated preference share for each attribute is the forecasted probability that each attribute is chosen as the most important [25]. Estimations were performed in NLOGIT 5.0 (Econometric Software Inc., Plainview, NY, USA). 

## 3. Results

Table 2 presents demographics for the 507 respondents who participated in the dog acquisition BWS question as well as two distinct subsamples: pet-owners, and non-pet owners. Although the total project sample (*n* = 1523) closely mirrored the U.S. census in terms of most demographics, the sample (*n* = 507) that was randomly selected to participate in the dog BWS experiment which is studied in this analysis had 15 percent less respondents from the Midwest and 14 percent more people from the South than the U.S. population. All demographics aside from geographic location of respondents were comparable in proportion within the sample to the U.S. population, according to U.S. Census data.

### 3.1. Level of Agreement Regarding Statements Related to Dog Acquisition

Statistically significant differences in agreement were seen amongst age categories and the income categories for the statement, “People should have choices as to where/how to obtain dogs.” The lowest percentage of respondents who selected agree were aged 18–34 (48.9%), followed by respondents aged 35–54 (56.8%) (Table 3). The largest percentage of respondents who selected agree were aged 55–88 (68.6%). A higher percentage of respondents who indicated an income of $51,000–$75,000 agreed with the statement “people should have choices as to where/how to obtain dogs” (52.6%) when compared to the other income groups. A higher percentage of respondents who indicated an income of $76,000–$100,000 selected disagree and a lower percentage of respondents who indicated an income of $101,000 or higher selected disagree when compared to the other income groups. One of the ways investigated as an option or choice for obtaining a dog was through sale/purchase. When presented with the statement, “The sale of dogs is socially irresponsible” a higher percentage of female respondents (31.1%) selected agree when compared to males (26.1%). Additionally, a higher percentage of respondents who indicated an income of $0–$25,000 selected disagree when compared to the other income groups. There was a higher percentage of residents from the South (30.4%) who selected agree to this statement, when compared to residents of the Midwest (24.3%).

For the statement “Dogs in pet stores come from irresponsible breeders”, a statistically higher percentage of women (39.8%) selected agree when compared to men (31.5%). A significantly higher percentage of respondents in the age groups 18–34 (30.4%) and 35–54 (32.6%) selected disagree in response to the statement when compared to the percentage of respondents in the age group 55–88 (23.4%).

In addition to investigating the sale/purchase of dogs in general, the sale/purchase of specifically purebred dogs was also studied (Table 4). In response to the statement, “People should be able to buy purebred dogs,” the lowest percentage of respondents who selected agree was the age group 18–34 (42.3%), followed by respondents aged 35–54 (54.9%). The highest percentage of respondents who selected agree was the age group 55–88 (64.4%). A higher percentage of respondents who indicated an income of $51,000 or higher selected disagree when compared to the other income groups. A significantly higher percentage of people who selected the West as their region of residence selected disagree in response to this statement when compared to people who selected the South and the Midwest as their region of residence. 

In response to the statement, “Importing of dogs for sale is irresponsible”, a higher percentage of female respondents selected agree (58%) when compared to men (45.5%). Respondents aged 55–88 (59.7%) had a higher percentage select agree when compared to the percentage of respondents aged 18–34 (45.3%) and 35–54 (48.2%). A lower percentage of respondents who indicated an income of $101,000 or higher selected disagree when compared to the other income groups. Few differences were found between the level of agreement with the statement, “The only responsible way to acquire a dog is through shelter/rescue” among the demographic variables studied. The only statistically significant (.05 level of significance) difference was that a higher percentage of women (39.6 %) selected agree than men (31.7).

A statistically higher percentage of women (57.6%) than men (47.5%) selected agree regarding the statement “There is a dog overpopulation problem in the U.S.”. A lower percentage of respondents that indicated an income from $0–$25,000 selected disagree (19.6%) than the other income categories. All age categories had statistically different responses with respect to the question “There is a dog overpopulation problem in the U.S.” Of those aged 18–34, 35–54, and 55–88, 42.3%, 52.6% and 59.9% selected agree respectively. Additionally, in respect to the question “There is a dog overpopulation problem in the U.S.”, a higher percentage of respondents who had not attained a college degree selected agree when compared to respondents who had a college degree or higher. 

A higher percentage of female respondents (41.6%) selected agree when presented with the statement, “Shelter dog populations would decrease if people stopped buying purebred dogs” when compared to men (35.9%). A lower percentage of respondents who selected the Midwest (33.0%) as their region of residence selected agree when compared to respondents who selected the South (40.7%) and the West (43.4%).

Only 40.4 % of men selected agree when presented with the statement, “Every shelter/rescue dog is adoptable” which was lower than the percentage of women (46.3%). A lower percentage of respondents who indicated an income of $0–$25,000 selected disagree (26.7%), and a higher percentage of respondents (40.3%) who indicated an income of $101,000 or more selected disagree when compared to the other income groups. A higher percentage of respondents residing in the West selected agree (48.5%) than did those who resided in the Northeast (38.8%) and South (41.2%). 

Regarding the statement “Importing of dogs for adoption is irresponsible”, a higher percentage of women, 51.0%, selected agree as compared to 42.8% of male respondents. A higher percentage (53.5%) of respondents aged 55–88 selected agree as compared to respondents aged 18–34 (39.4%), and those aged 35–54 (45.7%). 

### 3.2. Characteristics Rated of Importance for Dog Acquisition by Respondents

There were no statistically significant relationships between the rating of the characteristics behavior, and genetic health and any of the respondent′s demographics (Table 5). A lower percentage of respondents who reported an income of $0–$25,000 (54.4%) and a higher percentage of respondents who reported an income of $76,000–$100,000 (66.7%) stated breed was an important characteristic when acquiring a dog when compared to the other income categories. A higher percentage of men (67.5%) than women (61.1%) selected appearance as an important characteristic in acquiring a dog. However, a higher percentage of women selected important (88.8%) in regards to “compatibility with owner lifestyle” when compared to the percentage of men (82.5%). When compared to the percentage of respondents who reported the Northeast as their region of residence (84.4%), there was a higher percentage of respondents from the West (88.3%) who selected important with regard to the statement about owner lifestyle compatibility. 

Pertaining to the characteristic physical health, a lower percentage of respondents (79.9%) in the income category $0–$25,000 selected important when compared to the other income groups. Additionally, in regards to cost, a higher percentage of respondents aged 18–34 (71.8%) and 35–54 (68.0%) rated this characteristic as important when compared to respondents aged 55–88 (61.2%). A lower percentage of respondents who indicated an income of $101,000 or higher selected important in regards to the characteristic cost when compared to the other income levels.

Relative to “experience/reputation of the source”, a lower percentage of respondents who indicated an income of $0–$25,000 (68.5%) and a higher percentage of respondents with an income of $101,000 or higher (78.2%) selected important. Additionally, a higher percentage of respondents with a college degree or higher (75.7%) responded important when compared to respondents with less than a college degree (70.9%). A lower percentage of respondents from the Midwest and Northeast selected important when compared to respondents from the South. 

When presented with the characteristic, “source of the dog”, a higher percentage of women (71.3%) selected important when compared to men (66.4%). A higher percentage of people with at least a college degree (71.7%) selected important when compared to respondents with less than a college degree (66.7%). These differences in views of the importance of source of the dog necessitate further study into the perceptions of various demographic groups of various dog acquisition methods or pathways.

### 3.3. Multinomial Logit, Random Parameters Logit, and Latent Class Model Results

**Figure 1:**
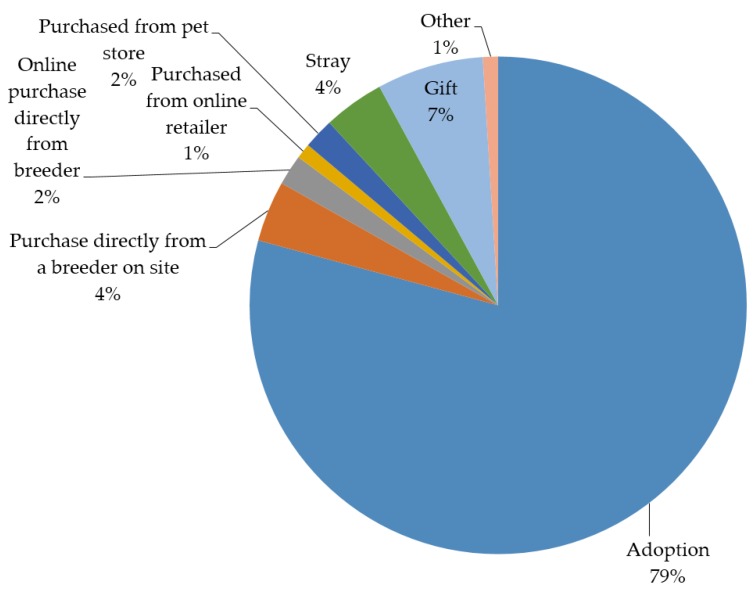
RPL preference shares for relative ranking of the perceptions of ethical acquisition methods by respondents.

**Figure 2:**
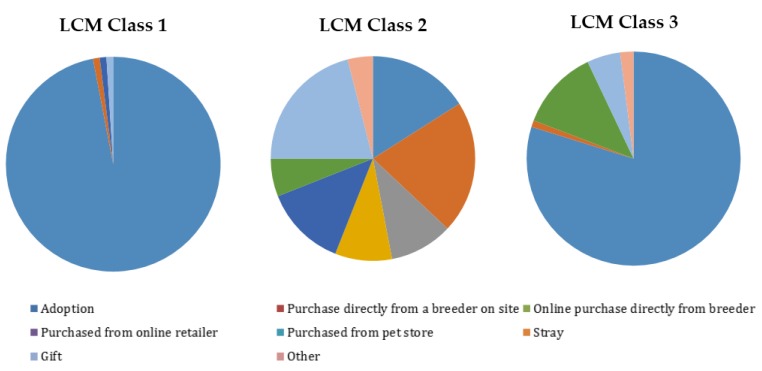
Preference shares for LCM classes.

The results of the MNL, RPL and LCM models are presented in Table 6, Figure 1 and Figure 2. All eight of the coefficients for the eight ways to acquire a dog were statistically significant for the MNL and RPL models. The standard deviations on each coefficient were statistically significant at the 5% level in the RPL model, suggesting heterogeneity as specified by the RPL model is an appropriate assumption for these preferences. The largest mean preference share from the RPL model was adoption with 80% of the mean preference share. The next largest mean preference share was gift as 6% of the preference share. The mean preference shares for purchased directly from a breeder on site, online purchase directly from a breeder, purchased from online retailer, purchased from pet store, stray, and other were all each less than 4%. These preferences do not indicate that the respondent has actually acquired a dog in this manner or will acquire a dog in this manner. However, these preference shares are indicative of a relative ranking of how ethical each method of acquiring a dog was perceived to be by respondents.

While the RPL model enables insight into respondents’ preferences and incorporates heterogeneity of preferences, the LCM enables the estimation of preferences for discrete segments of respondents which is often desirable for marketing or communications efforts. Preferences within a class of the LCM model are homogenous, while preferences across classes are heterogeneous. An LCM model was evaluated and the three class LCM model was determined most appropriate. Several covariates were analyzed to attempt to help characterize class membership, but only having acquired a dog by adopting in the past and gender (male) were statistically important to the analysis. Having acquired a dog by adopting in the past was a statistically significant predictor of class membership, decreasing the probability of membership for class two (relative to the other two classes). The covariate of having a male respondent was statistically significant and decreased the probability of membership for class two as well. 

The first class had a strong affinity toward adoption as the most ethical way to acquire a dog, so the class was dubbed “adoption only”. This first class had an overwhelming preference share of 96% for adoption. All other ways of acquiring a dog received less than 1% preference share for this group. Respondents were assigned membership to LCM classes, one, two and three based on the class for which they had the highest probability of membership. For example, if a respondent had 85% probability of being a member of class one, they were assigned to that class. After class assignment, demographic information was summarized by class in Table 7. The first class, “adoption only,” probabilistically contained the lowest number of respondents. All demographics were probabilistically evenly represented in this class.

The second class expressed more evenly distributed shares of preference amongst the various dog acquisition avenues than class one, so the class was referred to as “to each their own”. Obtaining dogs purchased directly from breeder on site and by gift, both tied as largest preference share for most ethical way to obtain a dog for this class, with 21% each. The next largest preference shares were for adoption (16%) and purchased from pet store (13%). All other ways to acquire a dog had shares of less than 10% each. As seen in Table 7, male respondents had a higher probability of class two membership. Fifty percent of all male respondents were probabilistically assigned to class two. Additionally, non-pet owners had a higher probability of class two membership. Forty-nine percent of non-pet owners were probabilistically assigned to class two. 

Class three appeared to have higher relative preference for homeless pets whether they were adopted or found, so the class was dubbed “Homes for the Homeless”. “Homes for the Homeless” had two primary acquisition methods at the top of those evaluated as most ethical (on the basis of preference share estimates). The preference share for adoption was 79% and the preference share for stray was 12%. All other ways to acquire a dog had preference shares of less than 5% each. As seen in Table 7, women had a higher probability of class three membership, with 50% of female respondents probabilistically assigned to class three. Additionally, lower income respondents were more likely to be probabilistically assigned to class three (43%). Pet owners were more likely to be probabilistically assigned to this class, with 48% of pet owners as class three members.

## 4. Discussion

In the current study, the most common differences seen (with regard to levels of agreement about statements related to dog acquisition) were between male and female respondents. More women agreed with the statements, “the only responsible way to acquire a dog is through shelter/rescue”, “there is a dog overpopulation problem in the U.S.”, “dogs in pet stores come from irresponsible breeders”, “shelter dog populations would decrease if people stopped buying purebred dogs”, “every shelter/rescue dog is adoptable”, “importing of dogs for sale is irresponsible”, “importing of dogs for adoption is irresponsible”, and “the sale of dogs is socially irresponsible”. These findings are in line with those indicating that women often show higher levels of concern for animal welfare than do men [26]. The pro-adoption stance of women is also consistent with reports by Markovits and Queen [27], that women predominate in dog rescue/sheltering and by Reese et al. [28], who noted that women were more likely to obtain dogs for rescue purposes and to get them from humane organizations such as shelters/rescues or from friends. This idea was further reflected by the class membership of women in the LCM. Women were more likely to be members of class three, whose highest preference shares for most ethical way to acquire a dog were for adoption, and stray.

Age was another important demographic associated with perceptions of various avenues for dog acquisition. The percentage of respondents aged 55–88 years was frequently statistically different to the other two age categories 18–34, and 35–54 years. A higher percentage of respondents aged 55–88 selected agree for the statements, “there is a dog overpopulation problem in the U.S.”, “people should be able to buy purebred dogs”, “people should have choices as to where/how to obtain dogs”, “importing of dogs for sale is irresponsible”, and “importing of dogs for adoption is irresponsible”. Those in the older age categories seemed more supportive of the idea that people should be able to choose where and which dogs they obtain (as long as those dogs were not imported from other countries). Similar age category differences have been observed relative to aspects of animal welfare examined in farmed animal species [29]. Some of these differences may be attributable to differences in experience as well as greater sensitivity of younger respondents to some dog welfare issues (e.g., high dog euthanasia rates in the U.S. and high numbers of dogs in shelters). Past studies have documented higher concern for animal welfare in younger people [26,30]. 

A greater proportion of older respondents selected disagree in regards to the statement “dogs in pet stores come from irresponsible breeders”. It is possible that at least some older respondents may have experienced sale of dogs in the local town pet stores as a familiar, normal practice and one that would not be permitted if breeders were irresponsible, than younger respondents. Similarly, the lowest percentage of respondents who agreed that people should be able to buy purebred dogs was in the age group 18–34 (42.3%). Higher concern for animal welfare has previously been reported in younger respondents [26,30]. Perhaps older respondents in the current study were less attuned to (or aware of) debates about potential concerns associated with sourcing dog from pet stores. In contrast, it is possible that older respondents were more sensitive to issues pertaining to importation of animals in general, as this would help to explain our current findings relative to their perceptions of statements related to dog importation.

Few studies have been conducted that provide a basis for understanding the results regarding the income demographic analysis. However, findings by Reese et al. [28], indicating that people with lower incomes (under $20,000) were more likely than those in other income groups to obtain dogs from sources other than breeders (family members or other people), suggest that they may be more open to acquiring dogs that have been previously homed or that they have not specifically purchased. This is in agreement with the finding that a higher percentage of respondents with an income of $76,000–$100,000 disagreed with the statement that people should have choices when acquiring a dog. Additionally, lower income respondents were more likely to be member of the LCM class three, “Home for the homeless”, which had higher preference shares for most ethical way to acquire a dog for adoption, and stray.

Education level also appeared to be a factor in source of acquisition and dog selection criteria. Respondents with college educations stated they were more likely to adopt from a shelter or rescue. Further, a higher percentage of those with at least a college degree selected important for the characteristics, “Experience/reputation of source” and “Source of the dog” when compared to the percentage of respondents with less than a college degree. This finding resembles that of Reese et al. [28], who found that respondents with college degrees were more likely to adopt from a shelter or rescue, suggesting that supporting humane organizations may be important to this demographic.

Several differences were observed between respondents’ views as a function of region of residence, some of which may be explained in part by cultural and/or social differences across regions. More respondents indicating their region of residence as the West disagreed that people should be able to buy purebred dogs, compared to those from the South and Midwest. The finding that a higher percentage of respondents from the South than the Midwest agreed that, “Shelter dog populations would decrease if people stopped buying purebred dogs” and, “The sale of dogs is socially irresponsible” may be a reflection of the higher level of commercial dog breeding (which supplies purebred dogs to pet stores and members of the public), that occurs in the Midwest than in other regions of the country [31]. Variation in regional companion animal and humane organization population dynamics [32], may correspondingly influence residents’ perspectives on issues such as pet overpopulation and ethical sourcing of dogs.

In regard to income, a greater percentage of respondents in the income category $76,000–$100,000 indicated breed as an important characteristic. Additionally, a lower percentage of respondents in the income category $101,000 or higher selected cost as an important characteristic, and a higher percentage selected experience as an important characteristic when compared to the other income groups. It is plausible that those with higher incomes may have more options available to them for obtaining dogs, supported by the finding that cost was not indicated as an important characteristic, and therefore can prioritize selection of them based on their physical health, breed and previous experience with, or the reputation of an entity providing dogs. This suggestion is further supported by the finding that a lower percentage of respondents in the income category $0–$25,000 selected physical health and experience as an important characteristic when compared to the other income categories. Additionally, previous studies found higher income groups were more likely to have obtained a dog from a breeder than those in lower income categories [28]. In contrast, those with lower incomes may be more sensitive to the expenses of dog ownership, and thus costs may be more influential in decisions about dog acquisition for these persons. 

In regards to the preference shares for dog acquisition, among the most important findings is that overall, the largest preference share for dog acquisition was adoption. This suggests that there has been effective translation of publicly disseminated messages promoting adoption as the ethical path to dog acquisition (relative to purchasing of dogs or obtaining them in other ways). Many of these messages and communications represent adoption as a more responsible option than the purchasing of dogs from stores and breeders, while the latter are often portrayed as commodifying dogs [28,33]. 

The different subgroups of respondents may be reflective of people’s perceptions of the ethics of dog acquisition. The first class, adoption only, may represent people who have strongly internalized messages pertaining to adoption or rescue of animals being the primary means of ethically acquiring a dog. It is plausible that respondents in this class are similar to those reported by Sinski [33] whose rationales for adoption/rescue are informed by increased awareness of the current high euthanasia rates of animals. If so, messages pertaining to dog rescue as a means of promoting positive social change [33] relative to animal welfare may be appealing to this group, whereas purchasing of dogs may not. Class three, whose preferences included dogs both from humane organizations and those that were found as strays, may have similar ethical rationales about dog acquisition to class one.

The second class, which had more evenly distributed preference shares for the different dog acquisition options may value consumer sovereignty or right to be able to make choices as described by Rippe [34]. Thus, while their highest preference shares were for purchasing dogs directly from breeders and receiving them as gifts, this group appeared not to have strong biases against obtaining dogs from many of the sources inquired about. Additionally, it is important to note that some pet stores have agreements with animal shelters and rescues to showcase and adopt out their animals. Respondents may be unsure if they are purchasing an animal bred for sale or adopting an animal in such situations. It is possible that these individuals have more, or different, experiences with obtaining dogs from varying sources, and therefore are not deterred by options that may be objectionable to members of the other subgroups. 

## 5. Conclusions

The findings of the current study affirm those of previous investigations reporting that factors such as appearance [4], compatibility with owner lifestyles, previous experience, and cost, inform people′s decisions about dog acquisition. The relative importance of each of these criteria varied depending on the demographics of the respondent, and as has been previously observed, women favored rescue/adoption more than men. Our results collectively indicate the growing appeal of adopting dogs from shelters and rescue organizations for many segments of respondents. Further exploration of the underlying values and beliefs of all of the sub-groups identified in this study is necessary. Additionally, further studies, targeting large samples of dog owners, may be able to make more direct connections between the stated preferences of individuals and their actual methods of acquiring dogs. Although these results only indicate respondents′ stated preferences, not their actual actions, understanding respondent’s self-reported views is an important first step to determining the basis for their views, and can indicate how they are communicating with others within their sphere of influence. Different segments of the companion animal industry and those with different dog interests may want to consider tailoring their education and/or marketing communications to these groups accordingly. 

## Figures and Tables

**Table 1:** Best-worst block example.

Example Attributes	Example Options	
	Most Ethical	Least Ethical
Gift from friend/family member		
Adoption (shelter or rescue organization)		
Stray		
Purchased from Pet store		
Online purchase directly from breeder (via the breeder′s website)		
Other (i.e., parking lot)		

**Table 2:** Respondent demographics (% of respondents).

Demographic Variable	Percent (%) of All Respondents	US Census	Percent (%) of Respondents Reportedly Owning a Pet	Percent (%) of Respondents Reportedly Not Owning a Pet
	*n* = 507		*n* = 316	*n* = 191
Gender	% Respondents			
Male	50%	51%	43%	62%
Age				
Under 18	0%		0%	0%
18–24	14%	13%	16%	10%
25–34	13%	18%	15%	9%
35–44	17%	17%	19%	13%
45–54	19%	19%	20%	18%
55–65	18%	16%	19%	15%
66–88	20%	17%	12%	34%
Number of adults in my family	2.09	-	2.13	
Number of children in my family	0.69	-	0.844	
Annual pre-tax household income				
$0–$25,000	24%	25%	23%	27%
$26,000–$50,000	25%	25%	23%	29%
$51,000–$75,000	16%	18%	19%	12%
$76,000–$100,000	14%	12%	16%	11%
$101,000 and Higher	20%	20%	20%	21%
Educational Background				
Did not graduate from high school	2%	2%	2%	1%
Graduated from high school, did not attend college	29%	30%	28%	31%
Attended college, no degree earned	28%	25%	31%	22%
Attended college, bachelor’s (B.S. or B.A.), associates or trade degree earned	28%	27%	26%	31%
Graduate or advanced degree (M.S., Ph.D., Law school)	13%	16%	12%	15%
Region of Residence				
Northeast	18%	18%	15%	23%
South	36%	22%	38%	34%
Midwest	23%	38%	21%	26%
West	22%	22%	26%	17%

**Table 3:** Cross-tabulations of level of agreement regarding statements related to dog acquisition and potential welfare aspects (% of respondents; *n* = 507).

Demographic	Gender		Age			Annual Pre-Tax Income					Education		Region of Residence			
	Male	Female	18–34	35–54	55–88	$0–$25,000	$26,000–$50,000	$51,000–$75,000	$76,000–$100,000	$101,000 and Higher	Less than a College Degree	College Degree+	Northeast	South	Midwest	West
The only responsible way to acquire a dog is through shelter/rescue																
Agree	31.7a	39.6b	32.4a	36.6a	37.3a	36.5a	33.8a	37.1a	35.7a	35.7a	35.6a	35.9a	35.1a	35.8a	33.0a	38.9a
Disagree	40.2a	34.9a	39.7a	36.0a	37.3a	34.7a	39.8a	34.2a	43.5a	37.3a	37.7a	37.2a	35.5a	37.2a	42.1a	34.9a
There is a dog overpopulation problem in the US																
Agree	47.5a	57.6b	42.3a	52.6b	59.9c	56.3a	53.0a	48.9a	52.2a	51.3a	54.9a	49.7b	48.2a	54.5a	53.8a	52.1a
Disagree	26.6a	21.3b	32.6a	24.3b	17.5c	19.6b	26.1a	27.2a	28.5a	20.8a	23.4a	24.6a	26.4a	23.4a	24.0a	22.6a
Dogs in pet stores come from irresponsible breeders																
Agree	31.5a	39.8b	33.1a	34.9a	38.3a	33.6a	34.3a	34.9a	36.6a	40.3a	34.8a	36.9a	37.3a	35.1a	34.8a	36.4a
Disagree	31.9a	25.1b	30.4a	32.6a	23.4b	29.6a	27.4a	30.5a	31.7a	24.4a	29.9a	26.4a	25.0a	27.7a	31.9a	28.9a
People should have choices as to where/how to obtain dogs																
Agree	58.1a	60.4a	48.9a	56.8b	68.6c	59.8a	59.6a	52.6b	55.9a	66.2b	59.9a	58.5a	59.1a	58.8a	59.9a	59.6a
Disagree	21.4a	18.6a	27.0a	22.0a	13.3b	16.7a	21.4a	23.5a	28.0b	14.3b	18.6a	21.7a	21.4a	20.4a	18.7a	19.3a
Every shelter/rescue dog is adoptable																
Agree	40.4a	46.3b	43.6a	44.5a	42.3a	45.2a	46.4a	43.8a	45.7a	35.7b	45.1a	41.2a	38.8a	41.2a	45.9a,b	48.5b
Disagree	32.8a	32.1a	31.1a	34.1a	31.9a	26.7b	29.3a	32.7a	37.1a	40.3b	30.3a	35.3b	37.0a	30.9a	33.0a	30.7a
Importing of dogs for adoption is irresponsible																
Agree	42.8a	51.0b	39.4a	45.7a	53.5b	24.4a	44.9a	46.0a	46.2a	51.9a	46.4a	47.9a	43.5a	49.2a	48.5a	44.6a
Disagree	31.7a	23.5b	34.8a	28.5b	21.6c	24.3a	28.8a	30.9a	32.8a	23.7a	28.0a	26.9a	29.3a	25.1a	28.1a	29.5a

Note: Statistically significant differences are indicated by differences in letters a, b, or c. A value labeled with an “a” is statistically different from a value labeled with a “b” or “c”, but not statistically different from another value labeled with an “a”.

**Table 4:** Cross-tabulations of level of agreement regarding statements related to dog acquisition through sale and potential welfare aspects related to dog acquisition (% of respondents; *n* = 507).

Demographic	Gender		Age			Annual Pre-Tax Income					Educational Background		Region of Residence			
	Male	Female	18–34	35–54	55–88	$0–$25,000	$26,000–$50,000	$51,000–$75,000	$76,000–$100,000	$101,000 and Higher	Less than a College Degree	College Degree or Higher	Northeast	South	Midwest	West
People should be able to buy purebred dogs																
Agree	53.8a	58.1a	42.3a	54.9b	66.4c	55.0a	57.0a	51.1a	53.2a	62.0a	55.7a	56.4a	53.6a	56.5a	59.9a	53.0a
Disagree	23.7a	19.1b	30.2a	23.9b	13.0c	18.0a	21.6a	26.1b	27.4b	17.2b	20.6a	22.3a	22.8a,b	20.1b	17.5b	26.2a
Shelter dog populations would decrease if people stopped buying purebred dogs																
Agree	35.9a	41.6b	39.7a	38.9a	38.1a	42.3a	38.0a	34.9a	40.3a	38.0a	38.7a	38.9a	36.6a,b,c	40.7c	33.0b	43.4a,c
Disagree	34.9a	30.5a	31.4a	34.1a	32.4a	28.6b	34.0a	35.3a	33.9a	33.1a	33.8a	31.3a	31.2a	33.2a	35.7a	30.1a
Importing of dogs for sale is irresponsible																
Agree	45.5a	58.0b	45.3a	48.2a	59.7b	52.4a	51.2a	48.5a	53.8a	53.9a	52.0a	51.7a	52.5a	50.3a	54.7a	51.2a
Disagree	29.0a	19.8b	29.9a	28.1a	17.2b	21.2a	26.4a	28.7a	29.6a	18.8b	24.7a	23.9a	26.4a	25.7a	24.0a	20.8a
The sale of dogs is socially irresponsible																
Agree	26.1a	31.1b	28.0a	31.0a	27.0a	31.2a	26.1a	30.5a	31.7a	25.0a	29.8a	27.1a	29.3a,b	30.4b	24.3a	29.5a,b
Disagree	45.2a	40.5a	43.6a	41.6a	43.3a	37.8b	46.2a	39.3a	48.9a	44.2a	41.3a	44.8a	43.1a	40.8a	46.2a	42.5a

Note: Statistically significant differences are indicated by differences in letters a, b, or c. A value labeled with an “a” is statistically different from a value labeled with a “b” or “c”, but not statistically different from another value labeled with an “a”.

**Table 5:** Cross-tabulations “Characteristics you believe are most important in acquiring a dog” (% of respondents; *n* = 507).

Demographic	Gender		Age			Annual Pre-Tax Income					Educational Background		Region of Residence			
	Male	Female	18–34	35–54	55–88	$0–$25,000	$26,000–$50,000	$51,000–$75,000	$76,000–$100,000	$101,000 and Higher	Less than a College Degree	College Degree or Higher	Northeast	South	Midwest	West
Breed	60.2a	56.2a	57.7a	60.1a	56.8a	54.4b	56.2a	58.8a	66.7b	60.7a	56.4a	60.5a	57.6a	56.7a	59.6a	59.6a
Appearance	67.5a	61.1b	65.0a	61.1a	66.4a	60.8a	62.8a	67.3a	66.1a	66.2a	63.5a	65.2a	64.1a	64.6a	66.1a	61.7a
Compatibility with owner lifestyle	82.5a	88.8b	87.1a	85.7a	84.7a	83.1a	85.8a	86.8a	83.9a	89.0a	84.6a	87.1a	84.4a,b	83.4b	88.0a,b	88.3a
Behavior	85.0a	87.4a	86.9a	86.5a	85.5a	84.1a	84.4a	87.1a	87.1a	89.6a	85.1a	87.7a	85.5a	84.8a	88.6a	86.7a
Genetic health	72.3a	72.9a	73.0a	69.7a	74.9a	79.1a	72.0a	75.4a	71.5a	74.7a	71.9a	73.6a	73.2a	70.3a	74.3a	74.4a
Physical Health	82.9a	84.9a	82.0a	84.0a	85.2a	79.9b	84.2a	84.2a	86.6a	86.7a	83.0a	85.1a	83.7a,b	81.7b	88.0a	83.7a,b
Cost	64.5a	68.2a	71.8a	68.0a	61.2b	67.2a	69.4a	67.6a	68.8a	59.1b	68.0a	64.3a	67.8a	65.3a	69.3a	64.2a
Experience/reputation of source	72.4a	73.5a	73.2a	71.5a	74.0a	68.5b	70.2a	75.0a	75.8a	78.2b	70.9a	75.7b	76.1a	69.1b	77.8a	72.0a,b
Source of the dog	66.4a	71.3b	72.5a	66.3b	68.6b	67.5a	66.2a	70.2a	72.6a	70.5a	66.7a	71.7b	71.7a,b	65.3b	72.5a	69.0a,b

Note: Statistically significant differences are indicated by differences in letters a, b, or c. A value labeled with an “a” is statistically different from a value labeled with a “b” or “c”, but not statistically different from another value labeled with an “a”.

**Table 6:** MNL, RPL, and LCM) results and derived preference shares for relative ranking of the perceptions of ethical acquisition methods by respondents. * indicates statistical significance.

Ways to Acquire a Dog	MNL	RPL			LCM					
					Coefficients			Share of Preferences		
	Coefficient	Coefficient	Standard Deviation	Preference Share	Class 1	Class 2	Class 3	Class 1	Class 2	Class 3
Adoption	2.9126 * (0.04738)	4.8834 * (0.11294)	2.96659 * (0.10013)	0.800169	9.15332 * (0.32080)	1.25752 * (0.07986)	3.65251 * (0.13912)	96%	16%	79%
Purchased directly from breeder on site	1.3312 * (0.04866)	1.85099 * (0.07713)	1.72174 * (0.07951)	0.038567	4.90618 * (0.22413)	1.5479 * (0.08261)	−0.57308 * (0.13591)	1%	21%	1%
Online Purchase directly from breeder	0.54001 * (0.04461)	0.92360 * (0.06654)	1.06672 * (0.05790)	0.015257	3.31155 * (0.19402)	0.829848 * (0.07517)	−1.61602 * (0.12239)	0%	10%	0%
Purchased from online retailer	0.19821 * (0.04223)	0.40993 * (0.06481)	1.23426 * (0.06016)	0.009128	2.17934 * (0.16754)	0.682548 * (0.07282)	−2.04044 * (0.11558)	0%	9%	0%
Purchased from pet store	0.76083 * (0.04607)	1.18906 * (0.07615)	1.87075 * (0.06587)	0.019895	4.19544 * (0.21984)	1.08038 * (0.08450)	−1.46145 * (0.13715)	1%	13%	0%
Stray	1.01191 * (0.04747)	1.95428 * (0.08184)	1.86076 * (0.07981)	0.042763	2.21052 * (0.13393)	0.323228 * (0.06891)	1.74034 * (0.13520)	0%	6%	12%
Gift	1.55953 * (0.04901)	2.42050 * (0.07054)	1.08132 * (0.07028)	0.068163	4.68639 * (0.23151)	1.53968 * (0.07899)	0.875457 * 0.(0.14428)	1%	21%	5%
Other	0.00000	0.00000	0.00000	0.006058	0.00000	0.00000	0.00000	0%	4%	2%
Constant					−0. 36065 (0.51328)	0.592083 (0.48724)	0			
Acquired a dog by adopting in the past					0.000153 (0.00032)	−0.00094 * (0.000373)	0			
Male					−0.13177 (0.26254)	−0.97995* (0.233144)	0			
Class Probability					0.206	0.386	0.408			

**Table 7:** Percentage of demographic categories probabilistically assigned to each LCM class.

Demographics	Class 1	Class 2	Class 3
	“Adoption Only”	“To Each Their Own”	“Homes for the Homeless Dogs”
	*n* = 103	*n* = 195	*n* = 209
Gender of respondent			
Male *n* = 254	17%	50%	33%
Female *n* = 253	23%	27%	50%
Age of respondents			
18–34 *n* = 133	15%	39%	46%
35–54 *n* = 182	16%	41%	43%
55–88 *n* = 192	28%	36%	36%
Income of respondents			
Lower income *n* = 334	20%	38%	43%
Higher income *n* = 173	21%	40%	39%
Education of respondents			
No college degree *n* = 298	21%	38%	40%
College degree *n* = 209	19%	39%	43%
Region of residence			
Northeast *n* = 92	24%	36%	40%
South *n* = 185	23%	37%	40%
Midwest *n* = 117	20%	39%	41%
West *n* = 113	13%	42%	44%
Respondent owns a pet			
Yes *n* = 316	20%	32%	48%
No *n* = 191	21%	49%	30%
